# 

**Published:** 1861-12

**Authors:** 


### CONTENTS.

ORIGINAL ESSAYS AND COMMUNICATIONS.

Calcium and its Compounds, ..... 629
Concerning Dental Literature and Dentistry in general, and Dr. Allen a little. By Wm. A. Pease ..... 641
Improvement in Rubber Plates. By J. A. M'Clelland, D. D. S. ..... 649

SELECTIONS.

The Prelimnary Education of Medical Students ..... 650
Dentition and its Derangements. By A. Jacobi, M. D. ..... 654
Remarks on the use of Tobacco. By D. J. Lyster, M. D ..... 660

CORRESPONDENCE.

Dentistry in California ..... 662

The Fourth Annual Session of the Indiana State Dental Association ..... 664

EDITORIAL.

